# Neural and mesenchymal stem cells in animal models of Huntington’s disease: past experiences and future challenges

**DOI:** 10.1186/s13287-015-0248-1

**Published:** 2015-12-14

**Authors:** Irina Kerkis, Monica Santoro Haddad, Cristiane Wenceslau Valverde, Sabina Glosman

**Affiliations:** Laboratório de Genética, Instituto Butantan, 1500 Av. Vital Brasil, São Paulo, 05503-900 Brazil; Hospital das Clínicas da Faculdade de Medicina da Universidade de São Paulo, 455 Av. Dr. Arnaldao, São Paulo, 01246903 Brazil; CellAvita Ltd, 1470 Al. Santos, 9th floor, block 909, São Paulo, SP 01418-100 Brazil; SoluBest Ltd, Weizmann Science Park, POB 4053 18 Einstein Street, Ness Ziona, 74140 Israel

## Abstract

Huntington’s disease (HD) is an inherited disease that causes progressive nerve cell degeneration. It is triggered by a mutation in the *HTT* gene that strongly influences functional abilities and usually results in movement, cognitive and psychiatric disorders. HD is incurable, although treatments are available to help manage symptoms and to delay the physical, mental and behavioral declines associated with the condition. Stem cells are the essential building blocks of life, and play a crucial role in the genesis and development of all higher organisms. Ablative surgical procedures and fetal tissue cell transplantation, which are still experimental, demonstrate low rates of recovery in HD patients. Due to neuronal cell death caused by accumulation of the mutated huntingtin (mHTT) protein, it is unlikely that such brain damage can be treated solely by drug-based therapies. Stem cell-based therapies are important in order to reconstruct damaged brain areas in HD patients. These therapies have a dual role: stem cell paracrine action, stimulating local cell survival, and brain tissue regeneration through the production of new neurons from the intrinsic and likely from donor stem cells. This review summarizes current knowledge on neural stem/progenitor cell and mesenchymal stem cell transplantation, which has been carried out in several animal models of HD, discussing cell distribution, survival and differentiation after transplantation, as well as functional recovery and anatomic improvements associated with these approaches. We also discuss the usefulness of this information for future preclinical and clinical studies in HD.

## Introduction

Huntington’s disease (HD) is believed to be due to a significant loss of medium spiny neurons in the brain. Main treatment efforts have, therefore, been focused on obtaining new medium spiny neurons to replace the damaged ones. One single transplantation of human fetal striatal tissue into brains of a small number of HD patients provided short-term improvement in both movement and psychological symptoms [1]. Tissue taken from aborted fetuses, however, offers only a very limited quantity of cells, which cannot be purified or improved. Therefore, alternative valuable sources, such as in vitro cultured, expanded and purified neural stem cells (NSCs)/precursor cells and mesenchymal stem cells (MSCs) are of great interest. In vitro models of HD have been developed and used in HD studies and in drug screening for HD [2]. It is hard to evaluate the effect of cell therapy in vitro, however, since it requires cell interaction of graft with host cells and tissues. The present review will provide a short description of HD degenerative nervous system disorder symptoms, causes, and current treatments, as well as recent achievements in animal studies employing NSCs/progenitor cells or MSCs in chemical and transgenic animal HD models in order to critically evaluate the use of the transplantation of these cells in HD treatment.

### Huntington’s disease

HD is an inherited, autosomal-dominant, neurodegenerative disorder that results from the expansion (36 or more repeats) of a sequence of three DNA bases, cytosine-adenine-guanine (CAG), within exon 1 of the huntingtin (*HTT*) gene [3–5]. CAG repeat length in the mutant allele accounts for approximately 70 % of the variability in age of onset of HD, while the number of CAG repeats in the normal allele does not modify the age of onset [6, 7]. Triplet repeat length also influences disease progression, even after controlling for age of onset [8]. HD affects all races [9] and shows a stable prevalence in most populations of white people, which is of about 5 to 7 affected individuals per 100,000 [10]. The mean age of onset of HD is approximately 40 years; however, the disease may occur from infancy to the ninth decade of life [11]. Median survival time varies between 15 and 20 years from onset [12].

Clinical features of HD include progressive motor dysfunction, cognitive decline and psychiatric disturbance, probably caused by both neuronal dysfunction and neuronal cell death [12]. Despite its widespread distribution, mutant HTT (mHTT) protein causes selective neurodegeneration and neuronal loss, which occur preferentially in the striatum and in deeper layers of the cortex at early stages of HD [13, 14]. In advanced stages of the disease, many other brain regions can be affected as well, such as the globus pallidus, thalamus, hypothalamus, subthalamic nucleus, substantia nigra and cerebellum [15–18]. Because of neurodegeneration, HD patients present typical involuntary movements called chorea (dance-like movements), manifested by spontaneous and transient muscle contractions [19, 20].

#### Huntington’s disease and neuronal cell loss

At a molecular level, HD is characterized by progressive loss of GABAergic medium spiny neurons, which constitute 95 % of all striatal neurons. As the disease progresses, neurodegeneration becomes most prominent in the neostriatum, commonly referred to as the striatum, which also includes the caudate nucleus and putamen. Striatal atrophy occurs in 95 % of HD brains, with a mean volumetric decrease of brain matter of 58 % [14, 21].

mHTT protein is thought to cause cellular dysfunction, neurodegeneration and associated clinical features primarily through a toxic gain of function [13]. Although the physiological role of normal HTT remains unidentified, many proteins are known to interact with HTT, such as brain-derived neurotrophic factor (BDNF), and this binding may be associated with HTT function [22]. HTT is normally expressed at highest levels in the brain, particularly in the cerebral cortex (layers II and V) and the striatum [23, 24]. HTT is also expressed in peripheral tissues, contradicting the restricted and regional pathology of HD [25]. HTT is mostly a cytoplasmic protein, though it is also found at low levels in the nucleus in both neuronal and non-neuronal cell types in HD [26–28]. Regarding mHTT, the pathogenic process associated with polyGln expansion may involve an interaction with other proteins or multimerization to build large insoluble aggregates in the striatum and the cortex of HD patients [14, 29–34]. Aggregates alter cell function by sequestering normal HTT [35], transcription factors [36], and transport proteins [37], ultimately leading to cell death. More recently, the accumulation of mHTT protein in the extracellular matrix in the brain of HD patients and in vitro spreading of these proteins from cell to cell have also been demonstrated [38, 39].

#### Huntington’s disease and brain-derived neurotrophic factor

The susceptibility of striatal neurons to atrophy in HD has been linked to nerve growth factors such as BDNF, which is a small dimeric protein expressed in the adult mammalian brain and has been shown to promote the survival of all major neuronal types and differentiation of striatal neurons [40–42]. The use of BDNF as a biomarker is still debated by the scientific community. Some reports show decreased levels of BDNF in the striatum and plasma of HD patients [43] while other studies show that *BDNF* gene transcription (mRNA) and protein plasma levels are variable in peripheral blood in HD patients and are not, therefore, good biomarkers for predicting HD onset [44]. However, experimental preclinical studies show that BDNF has an important role in neurodegenerative diseases [45–48]. As a neurotrophic factor, BDNF is vital for the growth and survival of neurons and glia. Thus, the promotion of endogenous BDNF upregulation may be key to neurodegenerative disease treatment [49]. Indeed, MSC transplantation into HD patients can serve as an alternative strategy to increase exogenous and endogenous BDNF expression [45–47], as has been shown, for instance, in subpopulations of human MSCs [50].

#### The immune system, inflammation and Huntington’s disease

A large body of evidence indicates that neuroinflammation has a pivotal role in the development of several neurodegenerative diseases [51, 52]. Yet the exact underlying inflammatory mechanisms and the definitive impact of the innate and adaptive immune systems in HD pathology are still not fully understood. Different reports have previously demonstrated peripheral immune system dysfunction in HD, including an increase in innate immune system plasma proteins, such as complement factors and cytokines, several of which are associated with disease progression [53–55]. Many of the inflammatory cytokines and chemokines found at elevated concentrations in HD patient plasma (mainly interleukin (IL)6, tumor necrosis factor (TNF) alpha and IL8), appear to originate from hyperactive monocytes [56, 57]. The pro-inflammatory cytokines IL6 and TNF are significantly increased in the striatum, plasma and cerebrospinal fluid in mouse models and in symptomatic as well as presymptomatic HD patients. This anomalous immune activation could be a target for future treatments aimed at slowing down HD progression [51, 52]. mHTT interaction with the key kinase of the nuclear factor kappa B (NFKB) pathway—the inhibitor of kappa B kinase—has been shown to be one of the causes of increased cytokine production in primary HD immune cells in humans, via increased activation of the NFKB signaling cascade upon stimulation with lipopolysaccharide. Elevated cytokine and chemokine levels found in HD patients correlate with disease progression and can be detected as early as 16 years before disease onset [54, 56, 58]. Patient blood cytokine composition and expression levels may be useful to establish the initial moment of therapeutic intervention. Patient blood signatures may also provide insights into the effects of HD on the brain, as well as serve as biomarkers of disease progression [59].

### Animal models of Huntington’s disease

It is of major concern that preclinical studies of neurodegenerative disease have failed to predict efficacy in the clinic. In some cases, this is a consequence of inappropriate use of the model system [60]. The models most frequently used in preclinical and academic studies are chemical and transgenic HTT fragment models, and most studies use chemical models for inducing HD, whereby HD-like symptoms are induced by quinolinic acid (QA) [61–67] or 3-nitropropionic acid (3-NP) [68, 69]. QA can be found endogenously, where it binds and activates the N-methyl-D-aspartate receptor, which is a glutamate receptor and ion channel protein found in nerve cells. At high concentrations it is neurotoxic by over-exciting the same receptors, eventually leading to neuronal cell death [70]. QA is used to induce neurodegeneration in animal models, including HD. 3-NP is also used to induce neurotoxicity via oxidative stress in striatum neuronal mitochondria. The effect is acute and variable and it depends on the animal, causing weight loss, lethargy, loss of motor control and atrophy in the striatum associated with neurodegeneration and death. Neither of these two chemical models reproduces the molecular events of neurodegenerative diseases and, in particular, of HD [71].

In preclinical studies of drugs for treating HT, the HTT fragment transgenic models are most widely used. These include mouse models such as N1T1-82Q2, R6/2, and R6/2-J2, all of which have a short mutated amino-terminal fragment of human HTT. These mouse models are all generated by the expansion in the CAG repeat of the first exon of HTT, causing symptoms similar to those observed in HD patients [62, 72] such as HTT aggregation, jerky movements and striatal atrophy [73]. The R6/2 andR6/2-J2 models have a well-characterized homogeneous phenotype and the advantage that it is possible to perform survival studies in a short time (3 months) [72]. N171-82Q mice have a longer HTT amino-terminal fragment than R6/2 mice, with 82 polyglutamines, and the N171-82Q phenotype is similar to, but less severe than, that of R6/2 and R6/2-J2 mice [74]. A variety of transgenic animal models of HD have been established and provide important insight into the pathogenesis of HD, but it is important to choose appropriate models in the specific case of evaluating the effects of stem cell transplantation. For instance, models that develop the disease quickly are adequate for short-term treatment studies, whereas genetic models that develop HD slower and for longer periods are best for the evaluation of long-term treatments. Complete information about HD animal models has already been published [75].

#### Stem cells in Huntington’s disease animal models

As mentioned above, one of the therapeutic approaches to HD is the use of stem cell-based transplantation. Here we discuss two main strategies of HD stem cell-based therapies: the use of NSCs/progenitor cells (Table 1) and the use of MSCs (Table 2). Generally, experimental protocols vary with regard to the HD animal model used, including differences in the origin of transplanted stem cells, the duration of in vitro stem cell expansion, the number of stem cell passages, expression of stem cell markers, cryopreservation, quantity of cells for transplantation, route of administration, time taken between transplantation and analysis, disease recovery, labeling and tracking of transplanted cells, evaluation of end-point of stem cell migration and differentiation after transplantation, and so on. Each of these aspects has their advantages and disadvantages, many of which are discussed in this review.

**Table 1:** Neural stem/progenitor cell transplantation in animal models of Huntington’s disease

Cells	Cell marker expression	Cell passage	Cell marker (visual)	Cell number and time of injection	Growth factor expression	Model/age	Time of analysis	Behavior/ striatal volume	Cell distribution/ survival	Cell differentiation	Cell migratory activity	Conclusions and references
Human fetal cortex stem cells (12 weeks post-conception)	Not specified	Neurospheres 12 weeks in culture	HN	200,000 cells; striatum 1 week after QA	CNTF+, CNTF–	QA rat	8 weeks post-graft	CNTF+ cells or CNTF– cells demonstrated significant improvement over the 8 weeks; increased striatal volume	Robust survival of HN and Ki-67-positive cells: striatum, GP, EPN, and SNpr	Co-localization of GFAP + HN in striatum only	CNTF– stronger migratory activity; GP, EPN, and SNpr	Striatal transplants of human fetal stem cells in HD rat QA model elicit behavioral and anatomical recovery [65]
Immortalized huNSC lines from fetal telencephalon tissue	ABCG2+, nestin+, vimentin+	No data	Lac Z	1 × 10^6^; right striatum 1 week prior to damage induction or 12 h after	BDNF secretion	3-NP rat	2 weeks post-graft	1 week prior to damage induction: significantly improved motor performance and reduced damage to striatal neurons. 12 h after: no improvement in motor performance	Striatum; robust survival	Positive for beta-tubulin III, GFAP, calbindin, GAD	Limited migration to graft core in striatum	Improved motor functions and reduced cellular damage, neurotrophic support by secreted BDNF. Differentiation of huNSCs to GABAergic neurons, but not cholinergic or dopaminergic neurons [69]
Immortalized huNSC line (15 weeks gestation)	Nestin+	~24 passages	Lac Z, BrdU	5 × 10^6^ cells; IV transplant; tail vein; 7 days post-QA	Not specified	QA rat	From 2 up to 8 weeks post-graft	Significantly greater striatal volume	Predominantly lesion side of hemisphere; additionally renal cortex, spleen and epithelium of bronchioles	BrdU+/GFAP+/NeuN+; BrdU+/parvalbumin–/DARRP-32–/calbindin–	3 weeks after : X-gal + cells in striatum: in the parenchyma and around vessels	Intravenously transplanted NSCs migrate to the lesion site, reduce cellular damage, and induce functional recovery. Differentiate into neurons and glia, NTD [63]
huNSCs: same as in Lee et al. [63]; 2n = 46, XX	Nestin+, vimentin+	~24 passages or more	Lac Z	1 × 10^5^ intraventricular; 10 × 10^5^ IV	Not specified	QA rat	3 weeks post-graft	No data	Predominantly lesion side of hemisphere	No data	From 2 to10 weeks X-gal + cells in striatum: in the parenchyma and around vessels	NSCs migrate into the striatum, from both ventricle or systemic circulation, NTD [64]
Immortalized mNSCs: MHP36 cells	Not specified	Not specified	PKH26	~400,000 cells; striatum	Not specified	3-NP rat	14 weeks post-graft	No effect on striatal volume	Predominantly populated areas of damage	Endogenous glial differentiation; PKH26 cell differentiation into astrocytes and neurons	Graft in the region of neuronal loss and striatum, no migration	MRI. Partial recovery of learning in water maze. No effect on striatal volume. Implanted cells did not penetrate through the glial scar to reconstruct lost tissue [68]
Allotransplant of striatal cells: a) neurospheres; b) cell suspension	Not specified	Neurospheres third to sixth passage	EGFP+	40,000 cells; striatum; 2, 7, and 14 days after QA	Endogenous BDNF expression stable before and after cell transplant	a,b) QA mice; c) R6/2 mice	14 days and 3 months post-graft	Not specified	a) 2 days after QA: significant graft survival	a) GFAP+ up to 3 months	Better migration of the cells in R6/2 versus QA	a) Best survival: combination of early transplantation + neurospheres
									b) 7 and 14 days after QA: reduced graft survival	b) Undifferentiated		b) Astroglia and microglia activation in the striatum after injection of QA After 3 months the graft volume was reduced [62]
									c) 3 to 4 weeks survival time			
Adult SVZ-derived rNPC	SOX2+	Neurospheres; suspension; passage not specified	BrdU-labeled cells	∼180,000 cells; striatum	Not specified	QA rat	8 weeks post-graft	Reduce functional impairment	∼12 % graft survival	GFAP+, NeuN+, DARPP-32+, GAD67+	Migrated extensively; striatum	Neural progenitor cell transplantation reduces rotational asymmetry and impairment of spontaneous exploratory forelimb use [66]
Embryonic LGE and MGE-derived rNSCs	Nestin+, GFAP+	Passage 2	PKH26, Hoechst, TOTO-3	100,000 cells; striatum	SCF	QA rat	3 or 8 weeks post-graft	Not specified	3 weeks	Undifferentiated	Striatum	SCF increased expression [61]
Adult SVZ-derived rNPCs pretreated with LiCl	SOX2+	Cultured in vitro 14 days before transplant	BrdU-labeled cells	∼150,000 cells; striatum; 21 days after QA	Not specified	QA rat	12 weeks post-graft	Acceleration of sensorimotor function recovery	Increased survival	GFAP+, NeuN+, DARPP-32+, GAD67+	Migration in striatum	LiCl priming did not alter the maximal distribution of NPCs across the striatum, while augmenting transplant efficiency and accelerating sensorimotor function outcome in vivo [67]

*3-NP* 3-nitropropionic acid, *BDNF* brain-derived neurotrophic factor, *BrdU* bromodeoxyuridine, *CNTF* ciliary neurotrophic factor, *DARPP-32* dopamine- and cAMP-regulated phosphoprotein, Mr 32 kDa, *EGFP* enhanced green fluorescent protein, *EPN* entopeduncular, *GABA* gamma aminobutyric acid, *GAD* glutamate decarboxylase, *GFAP* glial fibrillary acidic protein, *GP* globus pallidus, *HD* Huntington’s disease, *HN* human-specific marker to nuclear antigen, *huNSC* human neuronal stem cell, *IV* intravenous, *LacZ* beta galactosidase, *LGE* lateral ganglionic eminence, *MGE* medial ganglionic eminence, *mNSC* murine neuronal stem cell, *MRI* magnetic resonance imaging, *NPC* neuronal progenitor cells, *NSC* neuronal stem cell, *NTD* no tumorigenesis detected, *QA* quinolinic acid, *rNPC* rat neuronal progenitor cells, *rNSC* rat neuronal stem cell, *SCF* stem cell factor, *SNpr* substantia nigra pars reticulate, *SVZ* subventricular zone, *X-gal* 5-bromo-4-chloro-3-indolyl-β-D-galatopyranoside

**Table 2:** Mesenchymal stem cell transplantation in animal models of Huntington’s disease

Cells	Cell markers	Passage	Cell labeling	Cell number/inoculation site, inoculation time	Growth factor expression	Animal model/ age	Time of analysis	Animal behavior/ striatal volume	Cell distribution/ survival	Cell differentiation	Cell migratory activity	Conclusions and references
MSCs from mUCB	*Positive:*	Low: 3 to 8	Hoechst 33,358	400,000 cells per hemisphere	mRNA: BDNF	R6/2, 5 weeks mice		Significant differences were observed between R6/2 and high-passage mUCB MSCs at 10 weeks of age	Not specified	No differentiation	Not specified	Transplantation of low-passage mUCB MSCs did not confer significant motor benefits. Limb-clasping was not observed [122]
	CD45	High: 40 to 50										
	SCA1											
	SSEA4											
	MHC class I											
	MHC Class II											
rBM-MSCs	Not specified	Not specified	SPION	5 × 10^5^ or 1 × 10^6^; striatum	Not specified	QA rat	7 days after lesion	Not specified	Not specified	Not specified	Blood vessels and lateral ventricles in both hemispheres	Reduced brain damage and enhanced striatal expression of FGF-2 [125]
rBM-MSCs	Not specified	Not specified	Hoechst 33,258	200,000 or 400,000 cells per hemisphere; 28 days after 3-NP	mRNA: BDNF, collagen type I and fibronectin	3-NP rat	From 72 h to 14 days post-graft	Behavior improvements	No distribution	No differentiation	No migration	Increased mRNA:BDNF, collagen type I and fibronectin. Neuroprotective effect. Behavior improvement [126]
Human AT-MSCs; hypoxia	Positive: nestin, NG2, KDR, FLT1, and CD34	Not specified	Ad5-GFP	5 × 10^5^cells; bilateral striatum	mRNA: NGF, BDNF, bFGF, HGF, VEGF, IGF-1, GM-CSF, PDGF-α, EGF, CNTF	R6/2; 8.5 weeks mice	4 weeks after injection	Slowed behavioral deterioration	Not specified	Tuj-1 GABAergic neurons. PGC-1α master regulator of mitochondrial biogenesis increased in ASC treated mice	Limited	Reduced striatal degeneration and formation of ubiquitin-positive aggregates. Behavior improvement [123]
	Negative: neurofilament O4											
Human AT-ASCs; hypoxia	Same as above	Not specified	Vybrant DiO	10^6^ cells; striatum after injection of QA	Same as above	QA mice; 8.5 weeks	Same as above	Significant improvement in apomorphine-induced rotation tests	Not specified	BDNF, calbindin, GABA, GAD—neuronal enzyme	Near transplantation locus forming a lump	Neuroprotective effect. Behavior improvement [123]
Adult rBM-MSCs	Nestin+, GFAP+, SCF/c-kit+	Passage ≥10	PKH26, Hoechst and TOTO-3	100,000 cells; striatum		QA rat	3 weeks or 8 weeks post-graft	Not specified	Significant	Undifferentiated	Limited; striatum	SCF increased expression [61]
Human BM-MSCs	Positive: CD29, CD44, CD49c, CD49f, CD59, CD90, CD105, CD166	Early: 3 to 5	GFP-hMSCs	100,000 hMSCs; striatum	Not specified	N171-82Q mice	1, 3, 5, 7, 15, and 30 days post-graft	Decreased atrophy of the striatal volume	Survival: 15.1 % at 24 h; 4.5 % at 5 days; 0 % at 15 days	hMSCs are undifferentiated. Endogenous cell: NeuN, βIII tubulin	hMSCs recruit pre-existing neuronal cells to the striatum	Increased: FGF-2, CNTF, VEGF, NGF. Endogenous cell proliferation. Reduced striatal degeneration [96]
	Negative: CD34, CD36, CD117, CD45											
Human BM-MSCs; immortalized cell line	Not specified	Not applied	Bisbenzimide + TOTO3	200,000 cells per hemisphere	Not specified	WT mice	8 weeks post-graft	Not applied	Survival rate-significant	GFAP, DARPP-32	Human BM-MSC transplantation induces migration of endogenous neuroblast cells	Not specified [124]
Human BM-MSCs	Not specified	Not applied		200,000 cells per hemisphere	Not specified	QA mice	16 days post-graft	Improves: rotarod performance, striatum volume	Survival rate—significant. Reduced cell apoptosis	GFAP, NeuN, DARPP-32, F4/80 (macrophage and microglial marker)	Same as above	Neuroprotective effect. Behavior improvement. Reduced striatal degeneration [124]
Human BM-MSCs	Not specified	Not applied		200,000 cells per hemisphere	Not specified	R6/2-J2 mice	16 days post-graft	Improves: rotarod performance, striatum volume	Survival rate—significant. Reduced cell apoptosis	GFAP, NeuN, DARPP-32, F4/80	Same as above	Same as above [124]

*3-NP* 3-nitropropionic acid, *ASC* adult stem cell, *AT-ASC* adipose tissue-derived adult stem cell, *AT-MSC* adipose tissue-derived mesenchymal stem cell, *BDNF* brain-derived neurotrophic factor, *bFGF* basic fibroblast growth factor, *BM-MSC* bone marrow-derived mesenchymal stem cell, *CNTF* ciliary neurotrophic factor, *DARPP-32* dopamine- and cAMP-regulated phosphoprotein, Mr 32 kDa, *EGF* epithelial growth factor, *FGF-2* fibroblast growth factor 2, *GABA* gamma aminobutyric acid, *GAD* glutamate decarboxylase, *GFAP* glial fibrillary acidic protein, *GFP* green fluorescent protein, *GM-CSF* granulocyte-macrophage colony-stimulating factor, *HGF* hepatocyte growth factor, *hMSC* human mesenchymal stem cell, *IGF-1* insulin-like growth factor 1, *KDR* kinase insert domain receptor, *MHC* major histocompatibility complex, *MSC* mesenchymal stem cell, *mUCB* mouse umbilical cord blood, *NGF* nerve growth factor, *PDGF* platelet-derived growth factor (alpha polypeptide), *PGC-1α* peroxisome proliferator-activated receptor-γ coactivator 1 α, *QA* quinolinic acid, *rBM-MSC* rat bone marrow-mesenchymal stem cell, *SCF* stem cell factor, *VEGF* vascular endothelial growth factor, *WT* wild type

### Neural stem cells/progenitor cells

Fetal- or adult-derived NSCs/progenitor cells are considered an attractive source for cell therapy because they are already committed to neural differentiation. Primary cultures [62, 65] and fetus-derived immortalized cell lines [61, 63, 68, 69], as well as progenitor stem cells from central nervous system (CNS) brain tissues [66, 67], have been used in animal studies. NSCs/progenitor cells, when undifferentiated, express markers such as vimentin (present in migrating neural crest cells and in neural stem cells of the adult CNS) [64, 69], nestin (expressed predominantly in NSCs/progenitor neural cells) [61, 63, 64, 69], the transcription factor SRY (sex determining region Y)-box 2 (*Sox2*; known to be expressed at high levels in the neuroepithelium of the developing CNS) [66, 67], and other neuronal and glial markers, such as Abcg2 (ATP-binding cassette, sub-family G (WHITE), member 2) [69] and glial fibrillary acidic protein (Gfap) [61]. Such adult-derived NSCs/progenitor cells also express low levels of the major histocompatibility complex (MHC) class II antigens [76], and exhibit high survival rates when transplanted into normal adult rat brains [77, 78].

#### Neural stem cells/progenitor cells in vivo and the host immune system

Whether or not NSCs/progenitor cells, similarly to MSCs, exhibit therapeutic action—cell replacement and neuroprotection—the immunomodulatory effects of NSCs/progenitor cells still remain to be studied in depth [79]. Neuroprogenitor cells have a suppressive effect on T cells that is accompanied by a significant decrease in proinflammatory cytokines such as IL2, TNFα, and interferon-γ [80]. Moreover, NSCs/progenitor cells inhibit multiple inflammatory signals, as exemplified by the attenuation of T-cell receptor-, IL2-, and IL6-mediated immune cell activation and/or proliferation [81]. However, the transplantation of fetal NSCs/progenitor cells and embryonic stem cell-derived NSCs/progenitor cells into patients and in mice with Parkinson’s disease revealed an immune response [82, 83], which may be explained by the presence of microglia or astroglia in the primary cell suspension, which strongly express MHC class II molecules [84].

#### Routes of neural stem cell/progenitor cell transplantation

In the majority of studies carried out with HD models, NSCs/progenitor cells are transplanted directly into the striatum, where they show good survival and distribution predominantly in the damaged areas of the brain [61–63, 65–69]. However, these cells demonstrate limited migration in scar tissue [68]. In contrast, cells injected in the tail vein are associated with a wider brain distribution [63] and are found in the lesioned brain hemisphere, especially near blood vessels and in the parenchyma. Additionally, NSCs/progenitor cells are also found in peripheral organs, such as in the renal cortex, the spleen and the bronchiole epithelium [63] (Table 1). Intravenous (IV; systemic) administration of NSCs/progenitor cells also shows cell retention in lung capillaries directly post-injection, resulting in inflammation and apoptosis in lung tissue [85].

#### Neural stem cells/progenitor cells in Huntington’s disease animal models

NSC/progenitor cell transplantation has been carried out in several animal studies for treatment of HD, as summarized in Table 1. Different cell sources and preparation methods have been used: single cell suspension of primary culture of NSCs/progenitor cells [61, 62, 67] or neurospheres formed by NSCs/progenitor cells derived from brain tissue [62, 65, 66]. Several studies did not evaluate the expression of specific NSC/progenitor cell markers before transplantation into the animal model [62, 65, 68] (Table 1). The number of cells and cell passages and the cell labeling for tracing vary between the studies (Table 1).

Data on the capacity of NSCs/progenitor cells to differentiate into neurons in vivo are controversial; most studies demonstrate differentiation into neurons and glial cells [62, 63, 65–69], while a few report that transplanted cells remain undifferentiated [61, 62]. Glutamate decarboxylase (GAD)1 (also known as GAD67), is an important marker of neural differentiation in HD. It catalyzes the synthesis of gamma-aminobutyric acid (GABA), a neurotransmitter that promotes synaptogenesis and protection from neural injury. High GAD1 levels are, therefore, an important marker of recovery in HD patients. Parvalbumin and calbindin-D28k are calcium binding proteins expressed in GABAergic interneurons. The expression of these proteins was observed in four studies that used adult subventricular zone-derived rat NSC allotransplantation [66, 67] and immortalized fetal tissue-derived human NSC xenotransplantation [64, 69]. Expression of GAD1, calbindin-D28k and/or DARPP-32 (dopamine- and cAMP-regulated phosphoprotein, Mr 32 kDa) was observed in four studies [66, 67], while glial fibrillary acidic protein (GFAP) expression was seen in the majority of studies [62, 63, 65–67, 69] (Table 1). In one study [63], which used IV injection of immortalized cell line-derived human NSCs, expression of early markers of neuronal differentiation was found 2 months post-graft.

For successful therapeutic use of NSCs/progenitor cells in HD, it is likely that they need to differentiate into functional neuronal cells that can aid patient recovery. Differences in cell biodistribution between neurospheres, which do or do not express ciliary neurotrophic factor (CNTF), has been observed in HD models. Neurospheres that lack expression of CNTF demonstrate better migration activity in comparison with those that express this factor [65]. The migration ability of NSCs/progenitor cells in the transgenic HD R6/2 model differed from that in a QA chemical model [62]. Moreover, neuronal differentiation into GABAergic and dopaminergic neurons was only observed when using cell allotransplantation in the QA model [66, 67] as opposed to the genetic model. Interestingly, one study demonstrated that neurospheres show better graft survival compared with cell suspensions, but differentiate to GFAP-positive cells, usually astrocytes, instead of neuronal cells [62]. This information is relevant for future stem cell-based therapies and should be thoroughly verified.

An important issue regarding cell transplantation is the possibility of induction of tumorigenesis. Only two studies have tested the tumorigenic potential of NSCs/progenitor cells in normal animals and did not find any type of pathology. The absence of tumorigenic potential is an extremely important characteristic of NSCs/progenitor cells that are to be used therapeutically. Considering that NSCs/progenitor cells are frequently isolated from embryo/fetal tissues, which are immature and commonly associated with tumorigenesis, further studies are needed to guarantee that NSC/progenitor cell transplantation is not tumorigenic [63, 64].

Although NSC/progenitor cell transplantation has been shown to be sufficient to induce moderate functional and anatomical recovery in chemical HD models, with increased striatal volume, reduced cellular damage and partly induced differentiation of NSCs/progenitor cells into glial cells and neurons [66, 67], there are still limitations to their therapeutic use. These limitations include ethical concerns regarding the source of NSCs/progenitor cells as well as the low quantities usually derived from these sources, which hinder their use and reliability.

### Mesenchymal stem cells

MSCs are commonly found in bone marrow, umbilical cord and fat pads [86–88]. They are responsible for tissue regeneration in cases of disease or injury throughout life. This function of MSCs is mediated by self-renewal and plasticity (the ability to produce diverse types of differentiated cells). MSCs can be isolated from the aforementioned tissues and are easily cultured; after obtaining a small number of cells from a patient, they can be rapidly multiplied in vitro and cryopreserved for future clinical applications.

MSCs are believed to be ‘cellular paramedics’ since they secrete a variety of bioactive molecules, such as cytokines, which have ‘trophic activities’ that can promote a regenerative microenvironment, and other molecules that contribute to reconstruction as immunomodulatory mediators and even by carrying molecules into damaged cells [89]. Autologous and allogeneic in vitro expanded MSCs transplanted into the recipient organism migrate to the injury site in response to chemotactic stimuli, and also induce migration of intrinsic (endogenous) MSCs to the same site from the surrounding environment. MSCs can act by reducing chronic inflammation, inhibiting apoptosis and scar formation and stimulating mitosis of tissue-intrinsic progenitors, thus remodeling the damaged tissue [90]. Due to these properties, MSCs are known as ‘medicinal signaling cells’ [89, 91, 92].

MSCs also stimulate angiogenesis, the process of new blood vessel formation, which is closely linked to neurogenesis, the process by which new nerve cells are produced. Blood vessels play an important role as a framework for neuronal progenitor cell migration toward the damaged brain region. Paracrine factors secreted by MSCs also reduce the destructive effects of oxidative stress. Using all these mechanisms of action, MSCs can significantly improve lesioned microenvironments that lead to restoration of the damaged tissue [92–94]. According to recent publications, MSCs can repair neurodegeneration by secreting trophic factors and proteins that stimulate migration, differentiation and survival of intrinsic (endogenous) cells [89, 91, 92]. Among specific effects on nerve cells, these factors can promote axon extension, growth, and even cell attachment to substrate in vitro. Although there is evidence that MSCs promote cell growth and repair in the brain, it has not yet been definitively confirmed that MSCs can become mature nerve cells with the ability to signal, or communicate with, other nerve cells [94–96].

#### Mesenchymal stem cells in vivo and the host immune system

Many experiments have been carried out in which MSCs are transplanted into other organisms of the same or of different species. These cells are not rejected because MSCs have very low levels of MHC class II proteins and lack MHC class I proteins and cannot, therefore, present exogenous antigens to the recipient (host) organism [97–100]. As a result, they are perceived as endogenous. MSCs also interact with the host immune and inflammatory systems in other ways, as discussed below.

When human MSCs are labeled in order to track their migration and then injected into mice that have some type of tissue damage, they migrate evenly throughout the damaged tissues. These cells may or may not be present in the tissue for a substantial period of time, which depends on many factors, such as cell type, animal model, time of cell transplantation, and so on. The continued presence of MSCs is important, but not essential, in therapeutic treatments because it demonstrates the potential for positive long-term effects of transplantation. It is important to realize that the temporary presence of MSCs is not a result of the host immune response, since experiments in injured mice with and without functional immune systems yield the same results. Further investigations show that MSCs suppress the immune system and reduce inflammation [101]. In brain injury models, MSC treatment reduces the presence of microglia in the damaged brain and decreases the number of peripheral infiltrating leukocytes at the injured site by increasing anti-inflammatory cytokines [102]. In other words, MSCs can be transferred between organisms without eliciting immune rejection by the host, which renders them very good candidates for transplantation, immunosuppression and immunomodulation [100, 103–107].

#### Routes of mesenchymal stem cell transplantation and penetration through the blood–brain barrier

A crucial issue in cellular therapies for HD is the route of MSC delivery into the brain, which has been approached in a number of different ways. Several administration routes have been proposed to deliver MSCs into the CNS, such as intracerebral (hemisphere or more precisely striatum), intrathecal, IV, intrathecal plus IV into the space surrounding the spinal cord, and even intranasal [108, 109].

The blood–brain barrier (BBB) is formed in early embryological development through complex multicellular interactions between immature endothelial cells and neural progenitors, neurons, radial glia, and finally pericytes, which share similar features with MSCs. It selectively controls molecular and cellular trafficking between the bloodstream and brain interstitial space, which is a concern when considering routes of drug and cell delivery to treat brain malignancies and neurodegenerative disorders. Systemically infused MSCs may be able to treat acute injuries, inflammatory diseases, CNS stroke and even brain tumors because of their regenerative capacity and ability to secrete trophic, immunomodulatory, growth or other engineered therapeutic factors. However, whether MSCs possess the ability to migrate across the BBB in vivo under both normal and pathological conditions remains poorly resolved [110]. Systemic infusion (that is, IV) of in vitro expanded MSCs is a minimally invasive and convenient procedure that is used in a large number of ongoing clinical trials: acute graft-versus-host disease [111], acute myocardial function [112, 113], liver disease [114] and multiple sclerosis [115]. Therefore, it is essential to verify whether transplanted MSCs can home to and engraft at ischemic and injured sites in the brain in order to exert their therapeutic effects. During brain inflammation and injury, the BBB becomes compromised, allowing cellular trafficking through the BBB, including leukocyte trafficking to sites of CNS inflammation, as has been well studied and extensively reviewed [116, 117]. Several recent studies suggest that adipose tissue- and bone marrow-derived MSCs may possess leukocyte-like activities that enable them to interact with and migrate across the BBB after injury or inflammation [110, 118–120]. It is suggested that MSCs can transmigrate across the brain vascular endothelial monolayer through transiently formed inter-endothelial gaps [121]. Given that MSCs have this ability to transmigrate across the BBB, they can be administered IV, which is not as invasive as the intrathecal or intracerebral (for example, in striatum) routes [65].

#### Mesenchymal stem cells in Huntington’s disease animal models

The ‘simplicity’ with which MSCs can be obtained and cultured, as well as their unique trophic activities and the possibility of their transfer into the host without immune rejection, are the reasons why we are hopeful that MSCs may offer a promising way to develop treatments for neurodegeneration. Table 2 summarizes published data on MSC transplantation into HD animal models.

Note that none of the published HD animal studies used systemic (IV) MSC transplantation. Table 2 also shows the types of cells used as origin and pre-treatment. Allogeneic and xenogeneic, primary cultures and immortalized cell lines from bone marrow, adipose tissue and umbilical cord blood, grown under normal levels of oxygen (normoxia) and under low levels of oxygen (hypoxia), have been used [61, 96, 122–125].

The majority of cells used in vivo in HD models are referred to as MSCs, multipotent stromal cells or adipose tissue-derived stem cells. However, most published articles do not demonstrate that the cells used present the typical MSC immunophenotype in accordance with the minimal criteria for defining multipotent mesenchymal stromal cells, as established by The International Society for Cellular Therapy [95]. Snyder and co-workers [96] are the only ones who show that, among other cell markers, the cells used in their study also express CD90 and CD105, which are considered markers of MSCs. While only one study reported that mouse umbilical cord blood (mUCB)-derived MSCs do not express MHC class II cell surface molecules [122], other authors did not provide such information. A few publications report that the cells used express neurotrophic factor genes, but they do not clarify whether the products of these genes are translated into protein [61, 122, 126] (Table 2).

All studies used cells at passages no higher than 10, excluding one study, which used mUCB-derived MSCs at passages 40 and 50 [122]. Interestingly, these authors observed that the expression of pluripotent stem cell markers, such as SSEA4 (stage-specific embryonic antigen 4), increases with the passages, as well as that transplantation of high-passage mUCB-derived MSCs confers significant motor benefits compared with that of low-passage mUCB-derived MSCs. However, the use of MSCs from later passages is not usual in animal and clinical studies due to chromosomal instability.

Cell doses per transplantation and cell tracer use vary between studies. Each has their advantages and disadvantages, as discussed in the ‘Neural stem cells/progenitor cells’ section of this review; taken together, however, they confirm that MSCs reach and engraft into the damaged areas of brain in HD animal models. These methods also show that the cell graft is mainly restricted to the striatum—the cells are found near the transplantation site, forming a lump, and show no or very limited migration. In one study, the authors observed that the cells are mainly localized near blood vessels and lateral ventricles in both hemispheres [125]. The low migration capacity of MSCs can be partly explained by application of the cells directly to the injured site [127], which does not provide stimulus for their migration due to the inflammation process that ensues, which is very chemoattractive for MSCs. This, for example, has been shown in cell transplantation in muscular dystrophy in the golden retriever model, whereby, after intramuscular transplantation, MSCs do not migrate from the region of local muscle application [87].

Different chemical (QA and 3-NP) and genetic models (R6/2-J2, N171-82Q, R6/2) of HD have been used in MSC transplantation studies (Table 2). There is no standardization with respect to time interval between MSC transplantation and analysis of results (Table 2). The studies which analyze survival of MSC post-transplantation note short-term survival of transplanted cells and reduction of apoptosis of intrinsic cells [61, 96, 124]. Most authors report that transplanted cells remain undifferentiated post-graft [61, 96, 122, 126], which supports the statement that MSC activity is similar to that of CNS microvascular pericytes [128]. These latter cells have critical and complex inductive, structural, and regulatory roles, interacting with other cell types of the neurovascular unit, especially endothelial cells and astrocytes [110]. On the other hand, several studies demonstrate expression of neuronal markers in transplanted cells, such as Rbfox3 (RNA binding protein, fox-1 homolog 3, also known as NeuN), which is a neuron-specific nuclear protein; however, NeuN appears to be devoid of immunoreactivity towards cerebellar Purkinje cells [129].

In general, all studies carried out in HD animal models using MSC transplantation observed behavioral and memory improvements, reduced brain damage and amelioration of striatal degeneration, and enhanced expression of several striatal growth factors. Most authors attribute these results to the neuroprotective effect of MSCs (Table 2).

### Stem cells in Huntington’s disease clinical investigations

The prospect of using stem cells to intervene in neurodegenerative disease is promising. To date, however, only a small number of clinical trials has been undertaken, whereby fetal donor tissues have been transplanted into the striatum [130]. Cell therapies in HD are intended to protect neuronal populations that are susceptible to the disease and/or replace dysfunctional or dying neurons. Clinical progress in HD cell therapy has centered on establishing protocols for transplanting fetal-derived cells into the diseased striatum. This strategy is stimulating the development of stem cell therapy in the clinic and has been shown to provide patients with a period of several years of improvement and stability, but not with a permanent cure for the disease [131]. A long-term follow-up of patients over a 3- to 10-year postoperative period shows that fetal striatal allograft in HD is safe, although this study showed no sustained functional benefit [1]. The authors suggest that such a result is due to the small amount of cells that were grafted in this safety study compared with other reports of more successful transplants in patients with HD [1]. This obstacle can be overcome with new cell technologies, which allow stem cell in vitro expansion, while preserving their natural capacity for self-renewal and differentiation.

## Conclusion

**Fig. 1:**
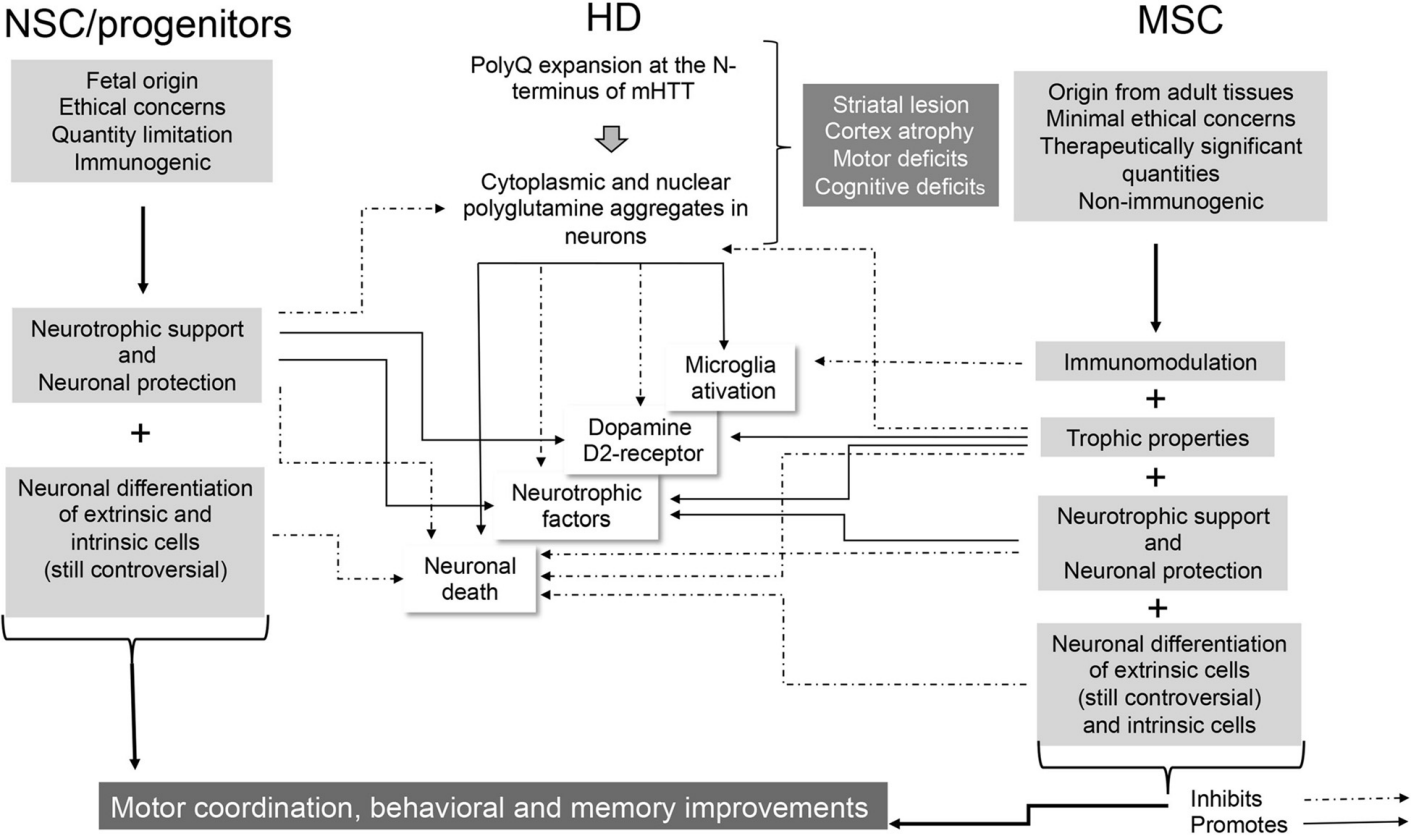
Effect of neural stem cells/progenitor cells and mesenchymal stem cell transplantation on Huntington’s disease etiology and progression. Huntington’s disease (*HD*) is caused by an expansion of (polyQ) repeats within the amino terminus of the huntingtin (*HTT*) protein, which promotes HTT aggregation and formation of intracellular inclusion bodies. These events lead to microglial activation, which correlates with striatal neuronal dysfunction and neuronal death as well as with reduced expression of striatal D1 and D2 receptors and of neurotrophic factors [136, 137]. In turn, striatal neuronal dysfunction correlates with cortex atrophy, motor deficits and cognitive deficits in HD patients. According to the most updated literature on HD, both neural stem cells (*NSCs*)/progenitor cells and mesenchymal stem cells (*MSCs*) improve motor coordination, behavior and memory. NSCs/progenitor cells and MSCs also seem to be able to reduce formation of HTT-ubiquitin aggregates. HD improvements occur as a result of NSC/progenitor cell and MSC transplantation through very similar mechanisms, such as immunomodulation, trophic properties, neurotrophic support and neuronal protection. These mechanisms are well known for MSCs and only marginally recognized for NSCs/progenitor cells [79, 94]. Until now, the great advantage of MSCs, in comparison with NSCs/progenitor cells, are their immunoprivileged properties, few or lack of ethical concerns regarding their origin, significant therapeutic quantities, non-teratogenicity (safety), as well as immunomodulation. Although in vivo differentiation of both cell types has been demonstrated, it is not clear if the number of differentiated cells is sufficient to justify all brain improvements found upon transplantation or whether changes are due to intrinsic cell regeneration. *mHTT* mutant huntingtin

The animal studies discussed in this review agree that NSC/progenitor cell and MSC transplantation can be beneficial, with partial functional and anatomical recovery, reduced cell death, reduced brain damage, increased endogenous cell proliferation and even partial differentiation of transplanted cells towards neurons (summarized in Fig. 1). More importantly, studies have even demonstrated reduced formation of ubiquitin aggregates upon adipose cell-derived MSC transplantation into HD mice [123] or when NSC therapy is associated with trehalose administration [132]. However, several points still need to be considered and answered using animal models.

It is worth mentioning that both the design of animal studies and the characterization of transplanted cells are poorly standardized and that this greatly complicates comparative analysis. In the future, an agreement between researchers must be reached in order to standardize marker use to enable study comparison and reproducibility.

It seems that NSCs/progenitor cells and MSCs can be used interchangeably. However, MSCs have an advantage over NSCs/progenitor cells in that there are fewer ethical considerations regarding their extraction and because they are easy to isolate and expand in vitro. Primary cultures of NSCs/progenitor cells are usually heterogeneous, containing many cell types, which makes characterization harder and experiments less reproducible. Moreover, MSCs are non-immunogenic, while neural stem/progenitor cells may require a co-application of an immunosuppression protocol (Fig. 1).

As to cell numbers (best dose) at transplantation, there does not seem to be any consensus. Fewer cells are probably best to avoid tissue damage upon transplantation. On the other hand, the population must be large enough to guarantee that sufficient cells can reach the area of tissue damage and promote recovery. Transplanted cells should be able to reproduce in the recipient organism while still undifferentiated, but their number should not be increased drastically in order to avoid carcinogenesis.

The administration route of stem cell transplantation should be revised, considering that local injections are extremely invasive and that NSCs/progenitor cells and MSCs do not show efficient migratory capacity, as extensively reviewed by Reyes and colleagues [133], among others [61, 87, 123, 126, 127, 133].

In chemical models, the cells are usually administered after HD induction with the drug, while, in transgenic animals, cell administration time depends on disease progression. Administration time should be adequately considered in order to derive the most benefit from the stem cell-based therapy.

So far, all animal and clinical study protocols for HD used only one course of cell transplantation. This is not compatible with the neurodegenerative character of the disease. In HD patients, the degenerative process is progressive and stem cell-based therapies should, therefore, be applied regularly. The point at which the therapy should begin and the time intervals between cell transplantations can vary significantly and are questions to be answered in future studies.

It is still unclear from animal studies how transplanted cells regulate the expression pattern of inflammatory cytokines and chemokines, as well as that of neurotrophic factors, which are also concerns that should be addressed before clinical trials.

Finally, HD therapy protocols using stem cells should be developed not only for treating the clinical onset of HD but also to prevent HD development [134]. The establishment of new methods to quantify mHTT in cerebrospinal fluid may facilitate the study of HD, since mHTT could potentially serve as a biomarker for the development and testing of experimental mHTT-lowering cell therapies for HD [135].

## Abbreviations

3-NP: 3-Nitropropionic acid
BBB: Blood–brain barrier
BDNF: Brain-derived neurotrophic factor
CNS: Central nervous system
CNTF: Ciliary neurotrophic factor
DARPP-32: Dopamine- and cAMP-regulated phosphoprotein, Mr 32 kDa
GABA: Gamma aminobutyric acid
GAD: Glutamate decarboxylase
GFAP: Glial fibrillary acidic protein
HD: Huntington’s disease
HTT: Huntingtin
IL: Interleukin
IV: Intravenous
MHC: Major histocompatibility complex
mHTT: Mutant huntingtin
MSC: Mesenchymal stem cell
mUCB: Mouse umbilical cord blood
NFKB: Nuclear factor kappa B
NSC: Neural stem cell
QA: Quinolinic acid
TNF: Tumor necrosis factor
